# Perceived importance of oral health among patients attending Katutura Hospital general outpatient department and dental clinic, Namibia

**DOI:** 10.2340/aos.v85.46511

**Published:** 2026-07-16

**Authors:** Juvenary John Rutabanzibwa, Andrew Ross

**Affiliations:** a Department of Community Dentistry, School of Dentistry, University of Namibia, Windhoek, Namibia; b Department of Family Medicine, Faculty of Public Health, University of KwaZulu-Natal, Durban, South Africa

**Keywords:** Dental clinic, Namibia, oral health, service utilization

## Abstract

**Objective:**

To examine determinants of oral health knowledge, perceived oral health status, perceived dental needs, and clinically assessed tooth decay among adults attending Katutura Hospital, Namibia.

**Material and methods:**

A cross-sectional hospital-based study was conducted among adults attending the dental clinic and general outpatient department. Of 660 invited participants, 639 were included in the final analysis (response rate 96.8%). Data were collected using structured questionnaires assessing socio-demographic characteristics, oral health knowledge, oral hygiene practices, perceived oral health status, and perceived dental needs. Clinical oral examinations were performed to assess tooth decay. Multivariable logistic regression analyses were conducted to identify factors independently associated with the outcomes.

**Results:**

Most participants (89.7%) demonstrated good oral health knowledge. Tertiary education was the only independent predictor of good oral health knowledge (adjusted odds ratio [AOR] = 2.47; 95% confidence interval [CI]: 1.54–3.97) and was associated with lower odds of tooth decay (AOR = 0.39; 95% CI: 0.27–0.57). Brushing twice or more daily was significantly associated with good self-rated oral health (AOR = 2.03; 95% CI: 1.45–2.84). Employment status was inversely associated with perceived dental needs (AOR = 0.71; 95% CI: 0.51–0.99).

**Conclusions:**

Education was the principal determinant of oral health knowledge and clinical tooth decay, while oral hygiene practices influenced perceived oral health. Strengthening oral health promotion and improving access to preventive services may reduce inequalities and unmet dental needs in Namibia.

## Introduction

Good oral health is essential for overall well-being, with implications for physical, social, and mental health, as it can affect not only teeth and gums but also the ability to eat and speak, as well as self-esteem. Poor oral health can lead to various problems, including tooth decay, gum disease, bad breath, and tooth loss and can be linked to more serious conditions such as heart disease and diabetes [1, 2].

The 2010/11 National Oral Health Baseline Survey in Namibia indicated that tooth extractions were the main services provided at government dental clinics due to many patients presenting late with advanced carious lesions. Although the government of Namibia has increased the number of district oral health facilities equipped with all the essential dental equipment from 14 to 27 and employed more oral health personnel, the high level of unmet treatment needs is a cause for concern [3]. It is estimated that of the population using these services, seven out of 10 Namibians (74%) have some form of dental disease/condition that needs attention [3].

Assessing an individual’s perceptions about the importance and seriousness of oral health is necessary to understand and predict individual utilization of dental health services and can be influenced by various factors. A cross-sectional study conducted in Romania on children aged from 7 to 18 years reported that their perception of oral health was influenced by physiological aspects such as body image, psychosocial aspects such as self-esteem, life satisfaction and quality of life, and functional aspects such as mastication [4]. The study also found that untreated tooth decay with pain was associated with the perceived need for treatment. However, these findings may not be generalized to the Namibian context, as the study was conducted in Romania, an Eastern European country. A study conducted in Tenali Mandal, East Coast of India, with a sample size of 1,500 that ranged in age from 5 to 74 years, found that 8% of people with decayed teeth did not seek help from health services as they had no pain. Although 34% of participants acknowledged that oral health was important, only 24.4% had visited a dentist in the past 6 months [5]. These findings highlight that the participants did not think that tooth decay was an important disease, or that maintaining good oral health was important for their general health, as they only sought treatment if the tooth decay was associated with pain or the loss of masticatory function.

Studies conducted in Africa have reported similar findings, with a Lesotho study on students aged 17 to 19 years finding that more than half lacked an awareness of the importance of oral health and had not visited a dentist in the previous year, despite having unmet treatment needs on clinical examination [6]. A study conducted in South Africa on the oral health-seeking behavior in the first 6 years of a children’s life found that cultural beliefs, costs, and attitudes of health professionals were determining factors in the choice of health services by parents for their children [7]. On assessing the effect of gender on perceived importance of oral health, a study on 97 males and females in Nigeria found no significant difference, the latter paying better attention to their oral health and perceiving their oral status to be better than the former [8].

Studies have explored the influence of socio-economic status on individual perception of the importance of oral health, with a study in Columbia on 998 pregnant women reporting that those with high socio-economic status and good education tended to have a more positive perception of oral health [9]. Similar findings in a study in Lithuania conducted in 2015 on 160 patients with acute dental infection highlighted that the higher social-economic population has better oral health status due to increased access to oral health services. Individuals who rated their oral health status as very poor tended to take it less seriously than those who rated it as very good [9, 10]. An oral examination of those who rated their oral status as poor revealed that the results were consistent with their perceived status. These studies suggest that the perceived importance of oral health may influence an individual’s health-seeking behavior and oral health status [7–9]. However, most of these studies were descriptive, with analytical data, which might have provided an in-depth understanding on why and how people perceive the importance of oral health differently, not being available.

Most patients seeking dental services in Namibia present with advanced oral lesions, including dental caries and gum diseases [3], which may lead to tooth loss, job and school absenteeism, reduced productivity, cause long hospital admissions due to infection, necessitate the use of expensive medications, and in extreme circumstances, result in death. These adverse effects can be prevented if interventions are done timely through raising oral health awareness by implementing oral health promotion and oral disease prevention interventions, including teeth restoration and oral hygiene instructions.

This study aimed to identify the factors that affect oral health among patients attending Katutura Hospital general outpatient department (OPD) and dental clinic.

### Research question/hypothesis

How does individual health-seeking behavior determine/influence utilization of oral health care services?

### Specific objectives

1. To understand the perceived knowledge of oral health among patients attending Katutura Hospital general OPD and dental clinic
2. To document the patterns of utilization of oral health services among patients attending Katutura Hospital general OPD and dental clinic as a surrogate marker for utilization of oral health services by the general population
3. To establish the association between perceived oral health and clinically assessed oral health.

## Materials and methods

### Study design

This cross-sectional study was facility based and entailed the collection of quantitative data using questionnaire surveys of patients attending the Katutura Hospital OPD and dental clinic from March to August 2024. This was done by documenting the patterns of oral health service utilization, assessing their oral health knowledge, understanding the perceived importance of oral health, and establishing the association between perceived oral health and clinically assessed oral health. The hospital is the second biggest referral hospital in Namibia and serves the Khomas Region with a population of approximately 486,186 people [11], as well as referred cases from other regions. Most of the patients who attend the OPD are walk-in or self-referred patients from around Windhoek, with an average of 112 patients per day. Katutura Hospital dental clinic is the biggest public sector dental clinic in Namibia, offering both general and specialized dental services, including oral and maxilla-facial surgery, such as tooth extraction, minor and major surgery, restorative dentistry (e.g. filling) and prosthodontics (e.g. dentures) and orthodontic appliances. The average number of patients seen at the dental clinic is 80 per day, with many being for tooth extractions, as the decay is so severe that restorative treatments are no longer possible.

### Inclusion criteria

The study population consisted of patients attending Katutura Hospital who were ≥18 years at the OPD and dental clinic from March to August 2024 and could speak and read English so as to be able to complete the questionnaire. The study population during this time consisted of 3,360 patients at the OPD and 2,400 at the dental clinic. Using the sample size calculation formula, the sample size was *N* = 3,360 (OPD) + 2,400 (dental clinic) *z* = value based on the confidence level of 95% 1.96, *d* = margin of error 0.05, and *p* = standard deviation 0.5. The desired sample size for OPD patients was 345, and the sample size for the dental clinic was 332 patients.

### Exclusion criteria

Patients were excluded who were not mentally fit, were clinically unstable, or refused to sign the consent form.

Systematic random sampling was used by selecting every second patient who met the inclusion criteria until the desired sample sizes of 345 in the OPD and 332 in the dental clinic were achieved. To ensure that every patient had an equal chance of being selected, every day the researcher picked one of two numbers out of a box, that being 1 or 2, and used this as a starting point for selection of participants. If number 1 was picked, then the odd-numbered (1st, 3rd, and 5th) patients in the queue were included, and if number 2 was picked, the first 20 even-numbered patients were selected (2nd, 4th, and 6th) each day. Those selected were interviewed by the researcher in a separate room for privacy and did not lose their place in the queue. In addition, those interviewed at the general OPD underwent a dental screening to assess their clinical dental needs, while those attending the dental clinic had a dental needs assessment done, with those requiring treatment being attended to as required.

### Questionnaire

Two data collection tools were used, the first being a quantitative questionnaire and the second a designed dental clinical record form to collect the dental screening data. The questionnaire consisted of closed-ended questions with Likert scale options from which the respondent had to select one (Appendix 1), these being administered by the researcher at both sites, which entailed introducing the study, obtaining consent, reading the questions and the response options, and completing the form. The questionnaire was developed from validated questions obtained from a similar study [6], as well as additional questions formulated by the researcher. The questionnaire consisted of four sections:

1. *Demographics:*
  - Gender: male, female
  - Age: NIDS-2016 age groups 18–19, 20–24, 25–29, 30–34, 35–39, 40–44, 45–49, 50–54, 55–59, and ≥ 60 years, dichotomized into young (≤40 years) and old (≥ 41 years)
  - Education: none, primary, secondary, and tertiary, dichotomized into lower (none, primary, secondary) and tertiary (college).
  - Employment level: unemployed and employed.
2. Perceived *knowledge of oral health:* A question with 5 Likert scale options (strongly agree*,* disagree, strongly disagree, and don’t know) was dichotomized into correct (strongly agree and agree) and incorrect responses (disagree, strongly disagree, and don’t know). Those who got incorrect answers were coded as poor, and correct answers were coded as good.
3. *Oral health practice and pattern of utilization of services*: 5 questions with Likert scale options from 3 to 5.
4. *Perceived oral health status*: Two questions with 5 Likert scale options from excellent to poor and very important to I don’t know coded from 1 to 5 and dichotomized into poor (fair and poor) and good (excellent, very good, and good) and important (very important, somewhat important, and not important (not important, not at all important, and I don’t know).
5. *Perceived dental treatment needs*: Two questions with 5 Likert scale: Low (low, very low, don’t know) and high (very high, high) and yes and no.

The clinically assessed data consisted of a checklist with dental caries status and treatment needs and periodontal status. Dental caries status was assessed by using the G.V Black classification, dichotomized, and coded 0 with no dental condition or unmet need and 1 with one or more dental conditions or needs. To obtain an acceptable level of reliability, examiners were trained and calibrated prior to the survey. To limit the confounding factors arising from inter-examiner and intra-examiner variations, the examiners received a 1-week training and calibration course from members of the Department of Community Dentistry, Faculty of Dentistry, University of Namibia. The Kappa values for the clinical caries diagnoses in the test-retest analysis were 0.84.

The two tools were piloted at Katutura Hospital with 10 patients selected randomly (5 from OPD and 5 from the dental clinic) a month before the study, and their comments were used to make changes to the questionnaire. The data analysis was done using IBM SPSS STATISTICS 30.0.0.0 computer software, with descriptive statistics consisting of three basic categories of measures: central tendency, variability (or spread), and frequency distribution. The raw data were entered into the computer by the researcher, and the Likert scale answers were transformed and re-coded into dichotomized variables to enable the data analysis by site (OPD and dental clinic). The association between the demographic variables and their knowledge and health status was determined using multivariable logistic regression analysis after adjusting for independent variables (age group, gender, education level, employment status, frequency of toothbrushing, and self-rated oral health status).

### Ethics and human subject issues

Ethical approval was obtained from the University of KwaZulu-Natal (HSSREC/00006068/2023) and from the research ethical committee of the Ministry of Health in Namibia (Ref: 22/4/2/3), and permission to conduct the study was granted by the management committee of Katutura Hospital. Study participants were required to provide informed signed consent to participate. They were informed that the anonymous research findings may be used to improve health service delivery in Namibia. Unique identifiers were used instead of participant’s names to ensure that confidentiality and anonymity were maintained throughout the study.

## Results

A total of 660 clients/study participants attending Katutura Hospital were invited to participate in the study, of whom 643 (97.4%) agreed to participate, with four (0.6%) being excluded during analysis due to being underage (below 18-year-old), giving a final response rate of 639 participants (96.8%). Of the 639 whose data were analyzed, 318 (49.7%) were recruited from the dental clinic and 321 (50.3%) from OPD.

### Demographic characteristics

Most participants were females at both the dental clinic (65.1%) and OPD (65.7%). Of the 639 study participants, more than half (61.5%) were between 18 and 34 years of age, and two-thirds were below 40 years of age. One third (*n* = 207, 32.4%) of participants had tertiary education level, nine (3.0%) had no formal education, the majority having a low level of education and being unemployed (Table 1).

**Table 1:** Participant’s demographic details.

Variable	Characteristic	OPD % (*N* = 321)	Dental % (*N* = 318)	Total % (*N* = 639)	Cumulative %
Gender	Male	110 (34.3)	111 (34.9)	221 (34.5)	34.5
	Female	211 (65.7)	207 (65.1)	418 (65.4)	100
Age group	18–19	12 (48.0)	13 (52.0)	25 (3.9)	3.9
	20–24	73 (56.2)	57 (43.8)	130 (20.3)	24.3
	25–29	61 (48.4)	65 (51.6)	126 (19.7)	44.0
	30–34	50 (44.6)	62 (55.4)	112 (17.5)	61.5
	35–39	39 (56.5)	30 (43.5)	69 (10.8)	72.3
	40–44	28 (46.7)	32 (53.3)	60 (9.4)	81.7
	45–49	14 (37.8)	23 (62.2)	37 (5.8)	87.5
	50–54	9 (33.3)	18 (66.7)	27 (4.2)	91.7
	55–59	17 (65.4)	9 (34.6)	26 (4.1)	95.8
	≥ 60	15 (56.6)	12 (44.4)	27 (4.2)	100
Age group Dichotomised	Young (≤ 40)	234 (49.1)	243 (50.9)	477 (74.6)	74.6
	Old (≥ 41)	87 (53.7)	75 (46.3)	162 (25.4)	100
Education level	None	9 (47.4)	10 (52.6)	19 (3.0)	3.0
	Primary	47 (56.6)	36 (43.4)	83 (13.0)	16.0
	Secondary	49.4(163)	167 (50.6)	330 (51.6)	67.6
	Teriary	102 (49.3)	105 (50.7)	207 (32.4)	100
Education Dichotomised	Lower	219 (50.7)	213 (49.3)	432 (67.6)	67.6
	Tertiary	102 (49.3)	105 (50.7)	207 (32.4)	100
Employment status	Employed	123 (38.8)	123 (38.8)	246 (41.3)	38.8
	Unemployed	198 (61.7)	194 (61.2)	392 (64.3)	100

OPD: outpatient department.

### Knowledge of oral health

66 (10.3%) of participants had poor oral health knowledge, while 573 (89.7%) had good oral health knowledge (Table 2). The demographic characteristics, knowledge of oral health, perceived oral health status, perceived oral health needs, and clinical assessment data of the participants at the dental clinic and OPD showed no statistically significant differences. These data sets were therefore combined and analyzed together to measure any association with the different variables. A multivariable logistic regression analysis test was conducted examining factors associated with the knowledge of oral health.

**Table 2:** Oral health knowledge.

Category	OPD *N* = 321 (%)	Dental *N* = 318 (%)	Total *N* = 639	%
Good knowledge	252 (50.9)	243 (49.1)	495	89.7
Poor knowledge	69 (47.9)	75 (52.1)	144	10.3

OPD: outpatient department.

Table 3 presents the findings of the multivariable logistic regression analysis assessing factors associated with oral health knowledge. Education level was significantly associated with oral health knowledge. Participants with tertiary education had 2.47 times higher odds of possessing good oral health knowledge compared to those with secondary education or lower (adjusted odds ratio [AOR] = 2.47; 95% confidence interval [CI]: 1.54–3.97; *p* < 0.001). Employment status did not show a statistically significant association with oral health knowledge although employed participants demonstrated higher odds of good knowledge compared to their unemployed counterparts; this association did not reach statistical significance (AOR = 1.46; 95% CI: 0.98–2.18; *p* = 0.063).

**Table 3:** Multiple logistic regression model for oral health knowledge.

Predictor variables	Categories	AOR (95% CI)	*P*
Age (ref: ≤ 40)	≥ 41	0.921 (0.596–1.424)	0.712
Gender (ref: male)	Female	1.310 (0.887–1.934)	0.174
Education (ref: secondary or lower)	Tertiary	2.467 (1.535–3.965)	< 0.001
Employment status (ref: unemployed)	Employed	1.463 (0.979–2.184)	0.063

AOR: adjusted odds ratio; CI: confidence interval.

The multivariable model was adjusted for age, gender, education level, and employment status.

Other variables, including age and sex, were not significant predictors of oral health knowledge.

Overall, after adjusting for all covariates included in the model, education level remained the only independent predictor of oral health knowledge.

### Oral health practice and pattern of utilization of services

The vast majority brush their teeth at least once a day, and just over half had seen a dentist in the previous year. The number of females (*n* = 23, 62.2%) who visited a dentist for a dental routine checkup was double that of the male participants (*n* = 14, 37.8%). However, this number was very small, as only 37 attended a dental clinic for routine checks (Table 4).

**Table 4:** Oral health practice and pattern of utilization.

Question	*N* (%) Responses				
What do you do when you have a dental problem?	Visit a dentist 340 (53.2)	Home remedy 207 (32.4)	Nothing 92 (14.4)		
How often do you brush your teeth?	Never 7 (1.1)	In the morning 190 (27.9)	Before bed 9 (1.4)	Twice a day 398 (62.3)	Every time I eat 35 (5.5)
How many dental visits did you do in the past year?	More than once 156 (24.4)	Once 182 (28.5)	None 301 (47.1)		
How often would you visit a dentist for routine check-up?	Once a year 110 (17.2)	Twice a year 189 (29.6)	Don’t know 340 (53.2)		
Reason for previous dental visit? Male Female	Pain 136 (35.0) 253 (65.0)	Filling 17 (35.4) 31 (64.56)	Cleaning 10 (25.6) 74.4 (29)	Routine check-up 14 (37.8) 23 (62.2)	Others 44 (34.9) 82 (65.1)

### Perceived oral health status

Table 5 shows the results of the multiple logistic regression model examining factors associated with perceived oral health status, perceived dental needs, and clinical assessed dental status (tooth decay).

**Table 5:** Multiple logistic regression model for perceived oral health status, perceived dental needs, and clinically assessed tooth decay.

Predictor variables	Categories	Perceived oral health status AOR (95% CI)	*P*	Perceived dental needs AOR (95% CI)	*P*	Tooth decay (clinically assessed dental status) AOR (95% CI)	*P*
Age (ref: ≤ 40)	≥ 41	0.841 (0.571–1.239)	0.381	0.750 (0.507–1.110)	0.150	0.799 (0.534–1.194)	0.273
Gender (ref: male)	Female	1.102 (0.728–1.437)	0.897	1.093 (0.776–1.539)	0.611	0.848 (0.598–1.204)	0.357
Education (ref: secondary or lower)	Tertiary	1.052 (0.728–1.520)	0.786	0.869 (0.602–1.254)	0.452	0.394 (0.273–0.569)	< 0.001
Employment status (ref: unemployed)	Employed	0.778 (0.557–1.089)	0.134	0.714 (0.511–0.997)	0.048	1.004 (0.713–1.414)	0.982
Frequency of toothbrushing (ref: ≤ 1 [incorrect])	≥ 2	2.027 (1.448–2.837)	< 0.001	—	—	0.085 (0.600–1.214)	0.379
Self-rating of mouth’s health (ref: Poor)	Good	—	—	—	—	0.548 (0.387–0.776)	< 0.001

AOR: adjusted odds ratio; CI: confidence interval.

The multivariable model was adjusted for age, gender, education level, employment status, frequency of toothbrushing, and self-rated oral health status.

After adjusting for potential confounders, toothbrushing frequency was the only variable significantly associated with perceived oral health status. Participants who reported brushing their teeth two or more times per day had significantly higher odds of rating their oral health as good compared to those who brushed once or less (AOR = 2.027; 95% CI: 1.448–2.837; *p* < 0.001). This indicates that individuals with more frequent brushing were about twice as likely to perceive their oral health positively.

In contrast, age, gender, education, and employment status were not significantly associated with self-rated oral health in the adjusted model (*p* > 0.05). Participants aged ≥40 years had lower odds of reporting good oral health compared to those aged ≤40 years (AOR = 0.841; 95% CI: 0.571–1.239; *p* = 0.381) although this was not statistically significant. Females showed slightly higher odds of reporting good oral health than males (AOR = 1.102; 95% CI: 0.728–1.437; *p* = 0.897), but the difference was negligible. Similarly, tertiary education was not associated with perceived oral health (AOR = 1.052; 95% CI: 0.728–1.520; *p* = 0.786).

Overall, the findings indicate that frequent toothbrushing is a key determinant of positive self-perceived oral health, whereas socio-demographic characteristics were not independently associated with perceived oral health status.

### Perceived dental needs

Table 5 presents the results of the multivariable logistic regression analysis examining factors associated with perceived dental needs. Employment status was the only variable significantly associated with self-rated dental needs. Employed participants were less likely to report high perceived dental needs compared to unemployed participants (AOR = 0.71; 95% CI: 0.51–0.99; *p* = 0.048). Other variables, including age, sex, and education level, were not significantly associated with perceived dental needs.

### Clinical assessed dental needs (tooth decay)

Table 5 shows the results of the multiple logistic regression model assessing factors associated with tooth decay based on clinical assessment needs.

Education level and perceived oral health were the only variables significantly associated with tooth decay after adjusting for other covariates. Individuals with tertiary education had substantially lower odds of having tooth decay compared to those with secondary education or lower (AOR = 0.394; 95% CI: 0.273–0.569; *p* < 0.001). This indicates a 61% reduction in the likelihood of tooth decay among participants with higher education, suggesting that education may play a protective role, possibly through better oral health knowledge, preventive practices, and access to dental services.

Similarly, participants who rated their oral health as good were significantly less likely to present with clinically assessed tooth decay than those who reported poor oral health (AOR = 0.548; 95% CI: 0.387–0.776; *p* < 0.001). This reflects 45% lower odds of tooth decay, indicating that self-perceived oral health status is a meaningful proxy for actual clinical outcomes. In contrast, age, gender, employment status, and toothbrushing frequency were not significantly associated with tooth decay in the adjusted model (*p* > 0.05).

## Discussion

This study examined determinants of oral health knowledge, perceived oral health status, perceived dental needs, and clinically assessed dental status (tooth decay) among adults attending Katutura Hospital. To assess the utilization of dental services, a population-based study would have been ideal, but due to the associated challenges, patients attending the General OPD and the dental clinic were used as surrogate markers for the general population. This method is consistent with other studies on health services utilization that have used hospital-based populations to represent the general population [11 –19]. The findings demonstrate that education was the principal socio-demographic determinant of oral health knowledge and clinical tooth decay experience while toothbrushing frequency influenced perceived oral health status and employment status was associated with perceived dental needs. Internal validity was addressed by using systematic random sampling, which ensured that all patients meeting the inclusion criteria had an equal chance of participating. To reduce information bias, the researcher established a good relationship and created a friendly environment with the participants to gain their trust and confidence to ensure the collection of accurate information. The researcher repeated the answers after each question to make sure that the participants agree with what was recorded.

### Knowledge of oral health

This study found that higher educational attainment was the only independent predictor of oral health knowledge. Participants with tertiary education were significantly more likely to have good knowledge compared to those with lower levels of education. Similar findings have been reported in sub-Saharan Africa, where education has been consistently linked to improved oral health literacy and preventive practices [20–22]. Higher education enhances the ability to access, understand, and apply health information and is often associated with better socioeconomic conditions and greater exposure to health promotion messages [20, 23].

In many African settings, including Namibia, oral health information is not uniformly integrated into primary health care and school curricula, which places individuals with lower educational attainment at a disadvantage [24]. Limited access to dental services, especially in rural areas, further restricts opportunities to acquire oral health knowledge [24, 25]. In contrast, age, gender, and employment status were not independently associated with knowledge. While some studies report gender differences favouring women [24], the present findings suggest that structural educational differences may outweigh demographic influences in this setting.

These findings underscore the need for targeted oral health education strategies for populations with lower educational levels. Community-based interventions, use of local languages, visual health messages, and integration of oral health into primary health care outreach could help reduce knowledge disparities and improve preventive behaviors.

### Perceived oral health status

Toothbrushing frequency was the only factor significantly associated with good self-rated oral health. Participants brushing twice or more daily were approximately twice as likely to report good oral health. This supports previous findings that oral hygiene behaviors are closely related to subjective oral health perception [26]. Individuals who adhere to recommended hygiene practices may experience fewer symptoms, reinforcing positive self-assessment. Gender was not a significant predictor of perceived oral health. Although women often report poorer self-rated health in general health studies, oral health perceptions in African populations appear to be influenced more by access to care and cultural norms than by gender differences alone [27]. Limited availability of preventive dental services and a treatment-oriented care system may result in similar experiences for both men and women, thereby reducing observable gender disparities in perceived oral health [27]. Other socio-demographic variables were not independently associated with perceived oral health status.

### Perceived dental needs

This study found that employment status was the only independent predictor of perceived dental needs. Employed participants were less likely to report higher dental needs than unemployed individuals. This may reflect better financial capacity among employed individuals to access dental services, purchase oral hygiene products, and seek preventive care. In many sub-Saharan African settings, including Namibia, oral health services are often paid out-of-pocket, making cost a major barrier to care for unemployed populations [23].

The lack of association between education and perceived dental needs contrasts with its strong relationship with oral health knowledge observed in other studies [20, 28]. This suggests that knowledge alone may not translate into perceived need for care when structural barriers such as cost, availability of services, and geographic access persist. Similar patterns have been reported in African contexts where service utilization is influenced more by affordability and access than by knowledge levels [28].

These findings highlight the importance of improving financial and physical access to oral health services in Namibia. Strengthening public dental services, integrating oral health into primary health care, and expanding outreach programs for unemployed and low-income populations may help reduce unmet dental needs.

### Clinical assessed oral health status (tooth decay)

This study identified education level and self-rated oral health as the only variables independently associated with clinically assessed tooth decay after adjustment for covariates. Participants with tertiary education had significantly lower odds of tooth decay compared with those with secondary education or lower. This finding is consistent with the well-documented social gradient in oral health, whereby individuals with higher educational attainment experience lower caries prevalence and better oral health outcomes [20, 29]. Education is a key determinant of health literacy, behavioral choices, and access to preventive services. Global evidence indicates that oral diseases disproportionately affect socially disadvantaged groups and that structural determinants, including education, play a central role in shaping exposure to behavioral risk factors such as high sugar consumption and inadequate preventive care [20, 30–32].

The protective association observed may reflect several pathways. Higher educational attainment is commonly associated with improved oral health knowledge, more consistent use of fluoridated toothpaste, healthier dietary practices, and greater utilization of dental services [32]. Education may also enhance problem-solving skills and health-seeking behavior, facilitating earlier intervention and reduced progression of carious lesions. These mechanisms are supported by previous epidemiological studies demonstrating inverse associations between education and untreated caries across diverse populations [21, 22]. The present findings therefore reinforce the importance of addressing social determinants within caries prevention strategies.

Perceived oral health status was also significantly associated with clinical tooth decay. Participants who reported good oral health had substantially lower odds of presenting with tooth decay. This suggests that self-perceived oral health status may serve as a meaningful proxy for objective clinical outcomes. Previous studies have demonstrated moderate to strong agreement between self-rated oral health and clinically assessed caries, periodontal disease, and tooth loss [33, 34]. Individuals tend to base their self-assessments on symptoms such as pain, visible cavities, and functional impairment. Although subjective, perceived oral health status appears to capture underlying disease burden and may be useful in large-scale epidemiological surveillance where clinical examination is not feasible.

In contrast, age, gender, employment status, and reported toothbrushing frequency were not independently associated with tooth decay in the adjusted model. The lack of association between toothbrushing frequency and tooth decay warrants consideration. While twice-daily brushing is widely recommended, frequency alone may not account for brushing technique, duration, fluoride concentration, or dietary sugar intake. Effective caries prevention depends on adequate fluoride exposure and reduced sugar consumption in addition to mechanical plaque control [20].

## Limitations

Several limitations should be acknowledged. The cross-sectional design precludes causal inference. Self-reported measures may be affected by recall or social desirability bias. Residual confounding from unmeasured factors, such as dietary sugar intake and fluoride exposure, cannot be excluded. Nonetheless, the study provides important evidence on the role of socioeconomic and perceptual factors in the distribution of dental caries.

## Conclusion

In conclusion, higher educational attainment and favorable perceived oral health were independently associated with lower odds of clinically assessed tooth decay. These findings support the need for public health strategies that address social inequalities and strengthen oral health literacy to reduce the burden of preventable dental diseases. To address this gap, the government needs to integrate oral health services into the existing Primary Health Care essential health services package to increase accessibility and raise awareness about the need to prevent the development of oral health problems. The findings can assist policy makers to develop targeted interventions to increase oral health services utilization and reduce complications resulting from advanced dental lesions among Namibians. In turn, this will improve the quality of life of many Namibians.

## Recommendations

Based on the findings of this study, several recommendations can be made to improve oral health knowledge, promote preventive oral health behaviors, and reduce the burden of untreated dental diseases in Namibia.

Firstly, oral health promotion programs should be strengthened and integrated into primary health care services. Education was the strongest predictor of both oral health knowledge and reduced tooth decay, indicating the importance of improving oral health literacy among populations with lower educational attainment. Community-based oral health education campaigns using simple language, visual materials, and local languages should be implemented, particularly targeting disadvantaged and low-literacy populations. Previous studies have shown that improved oral health literacy is associated with better oral hygiene practices, increased utilization of preventive dental services, and improved oral health outcomes [35, 36].

Secondly, oral health education should be incorporated into school health programs and community outreach activities. Early exposure to oral hygiene education may improve lifelong preventive behaviors such as regular toothbrushing and routine dental attendance. Collaboration between the Ministry of Health and Social Services and the Ministry of Education may facilitate the integration of oral health into existing health promotion initiatives. School-based oral health interventions have been shown to significantly improve oral health knowledge and oral hygiene behaviors among children and adolescents [37, 38].

Thirdly, preventive oral health services should be expanded and made more accessible, especially for unemployed and low-income populations. The findings showed that unemployed participants reported higher perceived dental needs, suggesting that financial barriers may limit access to care. Strengthening public oral health services, increasing outreach dental programs, and integrating preventive oral care into existing primary health care packages may help reduce inequalities in access to services. Socioeconomic inequalities in oral health service utilization have been widely reported in both developed and developing countries [20, 39].

Fourthly, there is a need to shift the current oral health system from a predominantly treatment-oriented approach toward prevention and early intervention. Many patients continue to seek care only when symptoms become severe. Public awareness campaigns should therefore emphasize the importance of routine dental check-ups, early treatment, and preventive care to reduce advanced dental disease and tooth loss. Evidence suggests that preventive and early intervention strategies are more cost-effective and contribute to improved long-term oral health outcomes [40].

Furthermore, oral hygiene promotion initiatives should encourage twice-daily toothbrushing with fluoridated toothpaste, as frequent toothbrushing was significantly associated with good perceived oral health. Health care workers, including nurses and community health workers, should be trained to provide basic oral health education during routine patient encounters. The use of fluoridated toothpaste and regular toothbrushing has consistently been associated with reduced dental caries and improved oral hygiene status [41, 42].

Finally, further research is recommended using community-based and longitudinal study designs to better understand causal pathways between socioeconomic factors, oral health perceptions, service utilization, and clinical oral health outcomes in Namibia. Future studies should also explore additional factors such as dietary habits, fluoride exposure, cultural beliefs, and barriers to accessing preventive dental services. Longitudinal and population-based studies are important for informing evidence-based oral health policies and planning [43].
